# Supplementary material for: Structures of human dual oxidase 1 complex in low-calcium and high-calcium states
Source: Nat Commun. 2021 Jan 8;12:155. doi: 10.1038/s41467-020-20466-9 (PMC7794343; doi:10.1038/s41467-020-20466-9)
Supplement: Supplementary file 1 — Supplementary Information [file 41467_2020_20466_MOESM1_ESM.pdf]

Supplementary Information for

Structures of human dual oxidase 1 complex in low-calcium and high-calcium states

This PDF file contains:

Supplementary Figs. 1-11

Supplementary Table 1

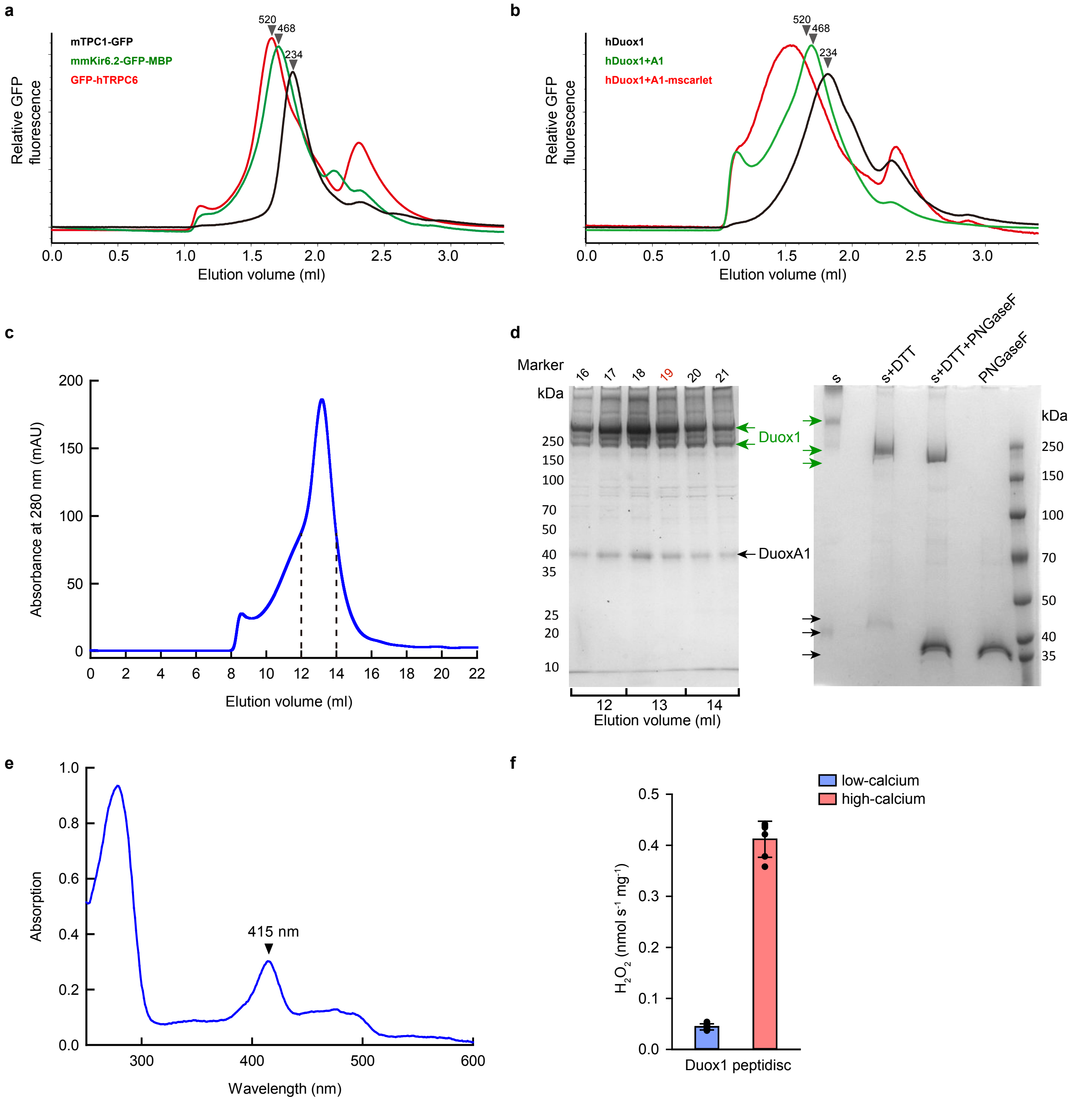

Supplementary Fig. 1 | Biochemical characterization of hDUOX1-hDUOXA1 complex. **a**, FSEC traces of three membrane proteins as molecular weight markers. mTPC1-GFP (234 kDa): mouse TPC1 channel with C-terminal GFP; mmKir6.2-MBP-GFP (468 kDa): Mus musculus Kir6.2 channel with C-terminal MBP-GFP; GFP-hTRPC6 (520 kDa): human TRPC6 with N-terminal GFP. **b**, FSEC traces of hDUOX1 and hDUOX1-hDUOXA1 complex. The positions of the molecular weight markers described in **a** are indicated by arrowheads. hDUOX1: hDUOX1 with N-terminal GFP; hDUOX1+A1: hDUOX1 with N-terminal GFP was coexpressed with nontagged hDUOXA1; hDUOX1+A1-mscarlet: hDUOX1 with N-terminal GFP was coexpressed with hDUOXA1 with C-terminal MBP and mscarlet. **c**, Size-exclusion chromatography of hDUOX1-hDUOXA1 peptidisc complex on a superose 6 column. The fractions indicated by dashed lines were used for SDS–PAGE. **d**, SDS–PAGE of hDUOX1-hDUOXA1 protein samples. SDS–PAGE (left) of the size-exclusion chromatography fractions labeled in **a**. Fraction 19 highlighted in red was concentrated for cryo-EM sample preparation. SDS–PAGE (right) of hDUOX1-hDUOXA1 protein samples treated with DTT or DTT+PNGase F. The positions of hDUOX1 and hDUOXA1 subunits are indicated by green and black arrows, respectively. The original gel was provided as a Source Data file. **e**, UV-Vis spectra of the purified hDUOX1-hDUOXA1 complex. The positions of the Soret peaks are indicated by arrowheads. **f**, Activity of the purified hDUOX1-hDUOXA1 complex peptidisc sample in the presence or absence of calcium. Data are presented as Mean ± s.d., n = 6 biologically independent samples. Source data are provided as a Source Data file.

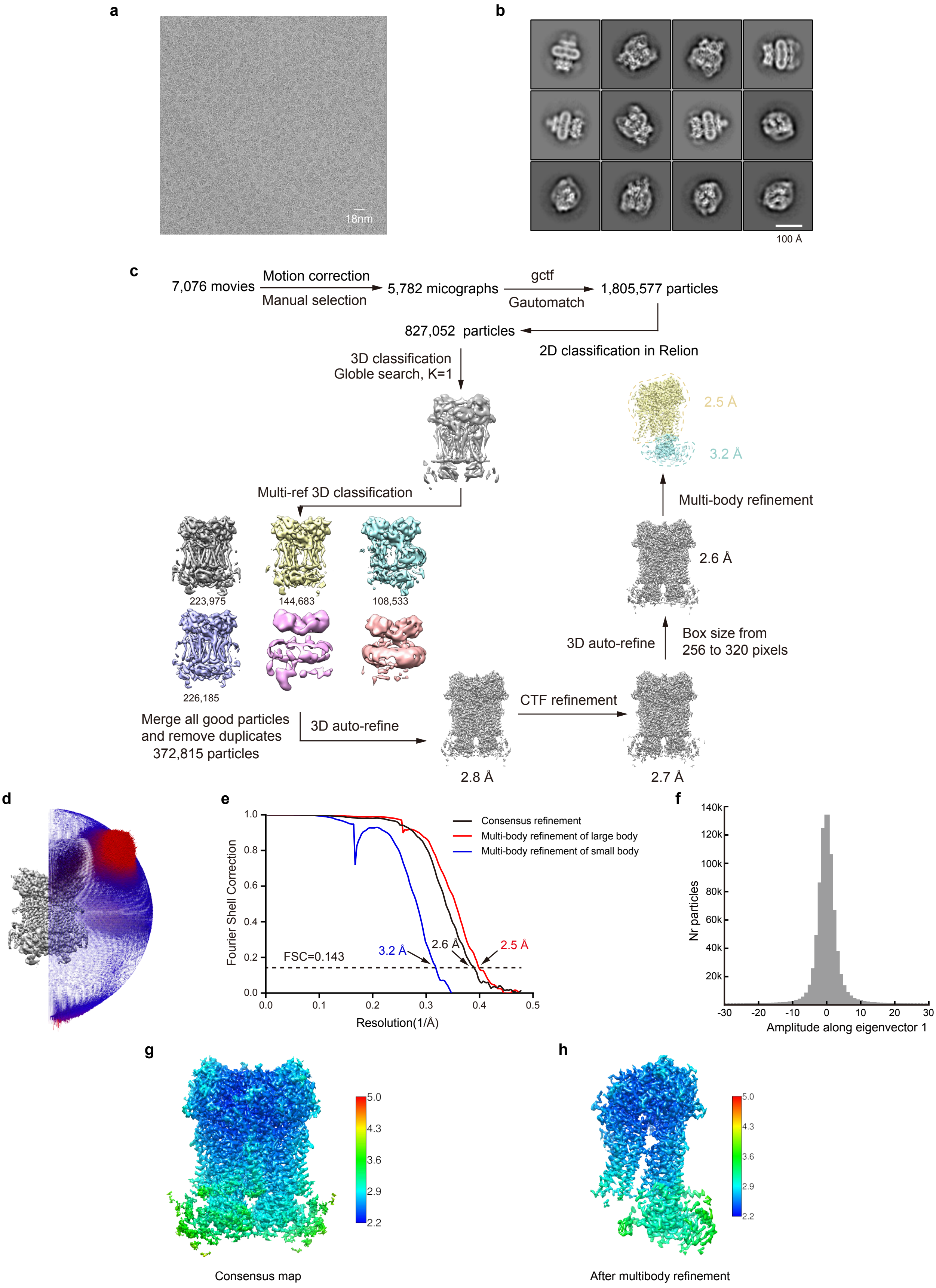

Supplementary Fig. 2 | Cryo-EM image processing of hDUOX1-hDUOXA1 complex in the high-calcium state. **a**, Representative raw micrograph of hDUOX1-hDUOXA1 in the high-calcium state out of 7,076 micrographs collected. **b**, Representative 2D class averages of hDUOX1-hDUOXA1 in the high-calcium state. Scare bar, 100 Å. **c**, The cryo-EM data processing workflow for hDUOX1-hDUOXA1 in the high-calcium state. **d**, The angular distribution for the consensus refinement of hDUOX1-hDUOXA1 in the high-calcium state. **e**, Gold-standard FSC curves of hDUOX1-hDUOXA1 in the high-calcium state. Resolution estimations are based on the criterion of FSC 0.143 cutoff. **f**, Histogram of the amplitudes along the top eigenvector shows monomodal distribution of hDUOX1-hDUOXA1 in the high-calcium state. **g**, Local resolution distribution of the consensus map of hDUOX1-hDUOXA1 in the high-calcium state. **h**, Local resolution distribution of the composite map of hDUOX1-hDUOXA1 in the high-calcium state after multibody refinement.

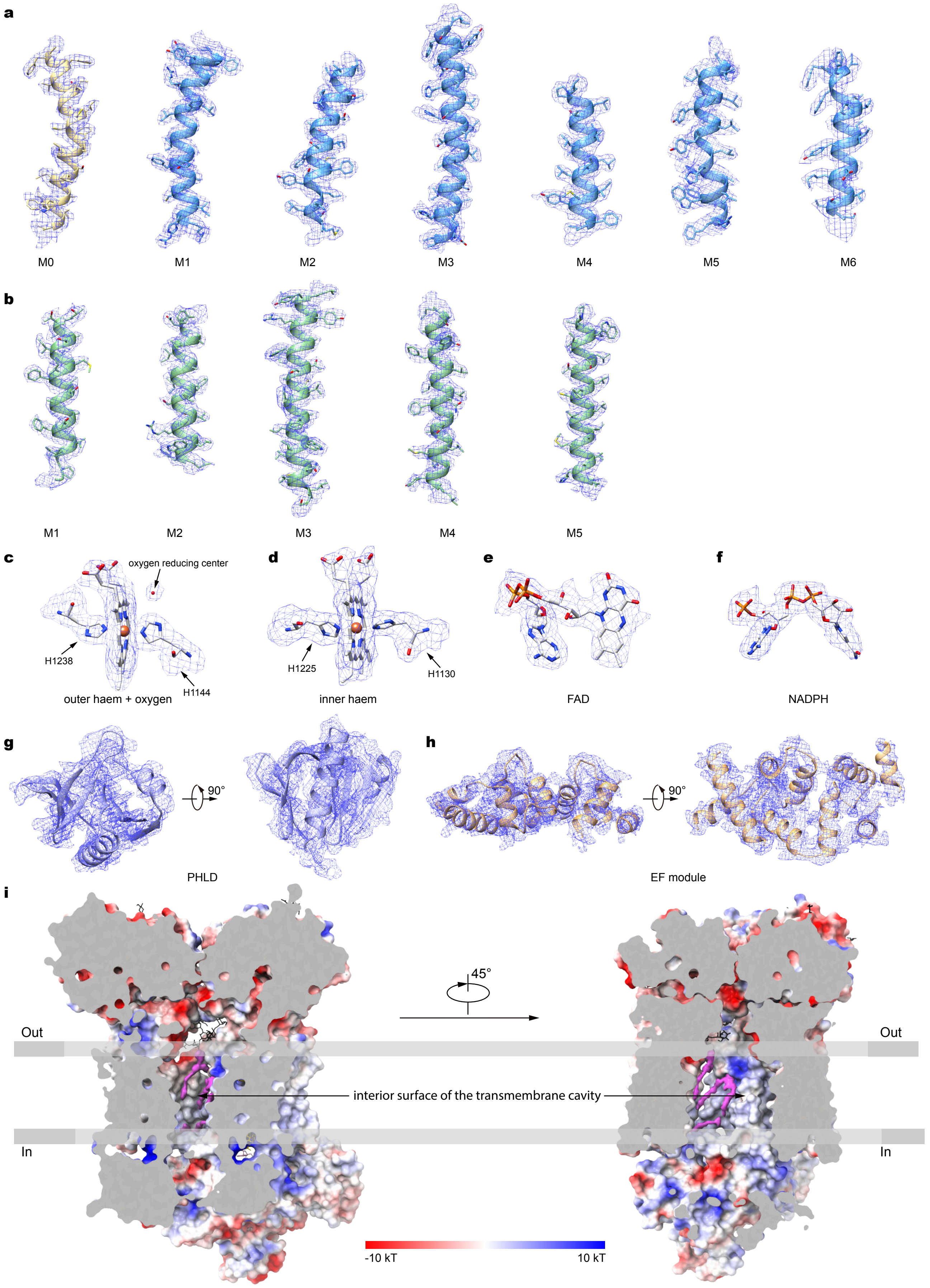

Supplementary Fig. 3 | Representative electron densities of hDUOX1-hDUOXA1 complex in the high-calcium state. a-b, Representative densities of transmembrane helices of hDUOX1(**a**) and hDUOXA1(**b**). **c-f**, Representative densities of the outer haem with the interacted histidines and spherical density at the putative oxygen-reducing center (**c**), the inner haem with the interacted histidines (**d**), FAD (**e**) and NADPH (**f**). **g-h**, Representative densities of PHLD (**g**), EF module (**h**). Models of hDuox1 and hDuoxA1 are colored the same as in Fig. 1**h**. The representative densities are all shown in blue mesh. Electron density maps are all contoured at σ=1.86. **i**, Cross section of the electrostatic surface representation of hDUOX1 shows a large highly hydrophobic central cavity in the transmembrane domain. The surface representation is colored according to electrostatic potential. Putative lipid densities on this surface are shown in magenta.

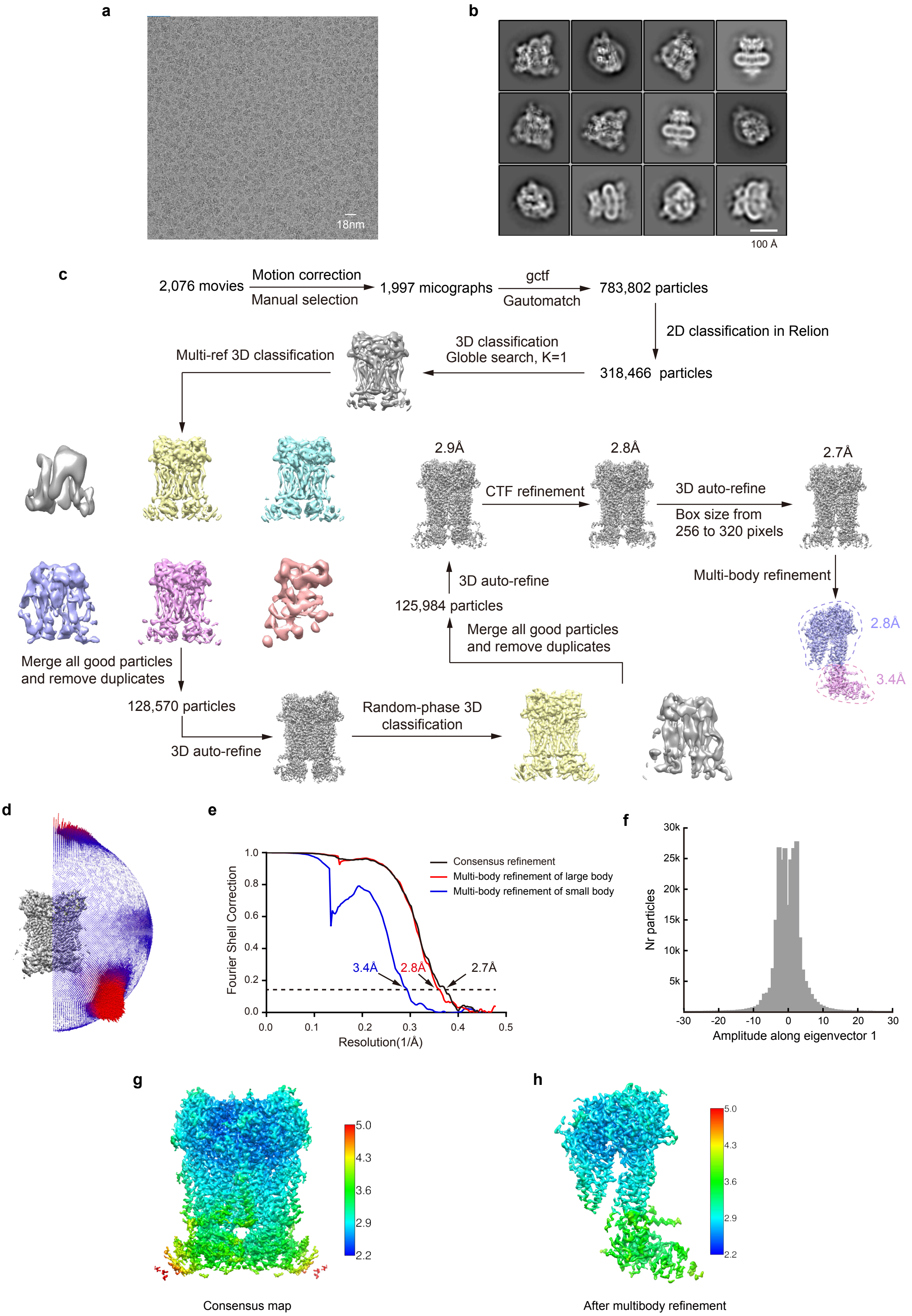

Supplementary Fig. 4 | Cryo-EM image processing of hDUOX1-hDUOXA1 complex in the low-calcium state. **a**, Representative raw micrograph of hDUOX1-hDUOXA1 in the low-calcium state out of 2,076 micrographs collected. **b**, Representative 2D class averages of hDUOX1-hDUOXA1 in the low-calcium state. Scare bar, 100 Å. **c**, The cryo-EM data processing workflow for hDUOX1-hDUOXA1 in the low-calcium state. **d**, The angular distribution for the consensus refinement of hDUOX1-hDUOXA1 in the low-calcium state. **e**, Gold-standard FSC curves of hDUOX1-hDUOXA1 in the low-calcium state. Resolution estimations are based on the criterion of FSC 0.143 cutoff. **f**, Histogram of the amplitudes along the top eigenvector of hDUOX1-hDUOXA1 in the low-calcium state shows a plateau-shaped distribution compared to that of the high-calcium state. **g**, Local resolution distribution of the consensus map of hDUOX1-hDUOXA1 in the low-calcium state. **h**, Local resolution distribution of the composite map of hDUOX1-hDUOXA1 in the low-calcium state after multibody refinement.

DUOX PHD

```
hDUOX1  1   MGF---------------------------------------------CLALAWTLLVGAWT-----PLGAQNPIS--WE 28
hDUOX2  1   MLRARPE-----------------------------------------ALMLLGALLTGSLG-----PSGNQDALSLPWE 34
drDUOX  1   MSV---------------------------------------------CLSTSLTLLFPNSTFILIFYLRAYSAIT--WE 33
dmDUOX  1   MSVPSAPHQRAESKNRVPRPGQKNRKLPKLRLHWPGATYGGALLLLLISYGLELGSVHC----------YEKMYSQ--TE 68
ceDUOX  1   MRSK-----------------------------------------HVLYIAILFSSIFG----------GKGIQQN--EE 27

hDUOX1  29  VQRFDGWYNNLMEHRWGSKGSRLQRLVPASYADGVYQPLGEPHLPNPRDLSNTISRGPAGLASLRNRTVLGVFFGYHVLS 108
hDUOX2  35  VQRYDGWFNNLRHHERGAVGCRLQRRVPANYADGVYQALEEPQLPNPRRLSNAATRGIAGLPSLHNRTVLGVFFGYHVLS 114
drDUOX  34  VQRYDGWYNNLADHDRGAADASLVRLYPAQYMDGVYLARQEPHLPNPRRISTTAMSGQSGLLSHKNRSVLSVAFGYHVWS 113
dmDUOX  69  KQRYDGWYNNLAHPDWGSVDSHLVRKAPPSYSDGVYAMAGA-NRPSTRRLSRLFMRGKDGLGSKFNRTALLAFFGQLVAN 147
ceDUOX  28  FQRYDGWYNNLANSEWGSAGSRLHRDARSYYSDGVYSVNN--SLPSARELSDILFKGESGIPNTRGCTTLLAFFSQVVAY 105

hDUOX1  109 DLVSVETPGCPAEFLNIRIPPGDPMFDPDQRGDVVLPFQRSRWDPETGRSPSNPRDPANQVTGWLDGSAIYGSSHSWSDA 188
hDUOX2  115 DVVSVETPGCPAEFLNIRIPPGDPVFDPDQRGDVVLPFQRSRWDPETGRSPSNPRDLANQVTGWLDGSAIYGSSHSWSDA 194
drDUOX  114 EISESRRAGCPPEFMHIKVQKDDPVFVSNSSQPVLLQFQRADWDTSTGKSPNNPRTQVNHVTAWIDGSSIYGSSSSWSDA 193
dmDUOX  148 EIVMASESGCPIEMHRIEIEKCDEMYDRECRGDKYIPFHRAAYDRDTGQSPNAPREQINQMTAWIDGSFIYSTSEAWLNA 227
ceDUOX  106 EIMQSNGVSCPLETLKIQVPLCDNVFDKECEGKTEIPFTRAKYDKATGNGLNSPREQINERTSWIDGSFIYGTTQPWVSS 185

hDUOX1  189 LRSFSRGQLASGPDPAFPRDSQNPLLMW-AAPDPATG-QNGPRGLYAFGAERGNREPFLQALGLLWFRYHNLWAQRLARQ 266
hDUOX2  195 LRSFSGGQLASGPDPAFPRDSQNPLLMW-AAPDPATG-QNGPRGLYAFGAERGNREPFLQALGLLWFRYHNLWAQRLARQ 272
drDUOX  194 LREFSGGRLSSSSSRDMPRRSSNGYLMW-SSPDPSSGPDSGSQELYEFGNAWANENIFSVTEGIIWFRYHNYLASKLHKE 272
dmDUOX  228 MRSFHNGTLLTEKDGKLPVRNTMRVPLFNNPVPSVMK-MLSPERLFLLGDPRTNQNPAILSFAILFLRWHNTLAQRIKRV 306
ceDUOX  186 LRSFKQGRLAEGVPGYPPLN-NPHIPLNNPAPPQVHR-LMSPDRLFMLGDSRVNENPGLLSFGLILFRWHNYNANQIHRE 263

hDUOX1  267 HPDWEDEELFQHARKRVIATYQNIAVYEWLPSFLQKTL--PEYTGYRPFLDPSISSEFVAASEQFLSTMVPPGVYMRNAS 344
hDUOX2  273 HPDWEDEELFQHARKRVIATYQNIAVYEWLPSFLQKTL--PEYTGYRPFLDPSISPEFVVASEQFFSTMVPPGVYMRNAS 350
drDUOX  273 HPSWSDEELFQHARKRVIATFQMAALV--FFSLL---------AGYQKYVDPGISVEFEAAAVRFGLTLAPPGVYKRNRT 341
dmDUOX  307 HPDWSDEDIYQRARHTVIASLQNVIVYEYLPAFLGTSL--PPYEGYKQDIHPGIGHIFQAAAFRFGHTMIPPGIYRRDGQ 384
ceDUOX  264 HPDWTDEQIFQAARRLVIASMQKIIAYDFVPGLLGEDVRLSNYTKYMPHVPPGISHAFGAAAFRFPHSIVPPAMLLRKRG 343

hDUOX1  345 CHFQGVINRNSSVSRALRVCNSYWSREHPSLQSAEDVDALLLGMASQIAEREDHVLVEDVRDFWPGPLKFSRTDHLASCL 424
hDUOX2  351 CHFRKVLNKGFQSSQALRVCNNYWIRENPNLNSTQEVNELLLGMASQISELEDNIVVEDLRDYWPGPGKFSRTDYVASSI 430
drDUOX  342 CHYRSVVNDDASKSPGLRLCNTFWNRNNPHLQSSLDVDELIMGMASQIAEREDNIIVEDLRDYMYGPLRFSRSDAVALTI 421
dmDUOX  385 CNFKET----PMGYPAVRLCSTWWDSSGF--FADTSVEEVLMGLASQISEREDPVLCSDVRDKLFGPMEFTRRDLGALNI 458
ceDUOX  344 NKCEFR--TEVGGYPALRLCQNWWNAQDI--VKEYSVDEIILGMASQIAERDDNIVVEDLRDYIFGPMHFSRLDVVASSI 419

hDUOX1  425 QRGRDLGLPSYTKARAALGLSPITRWQDINPALSRSNDTVLEATAALYNQDLSWLELLPGGLLESHR-DPGPLFSTIVLE 503
hDUOX2  431 QRGRDMGLPSYSQALLAFGLDIPRNWSDLNPNV---DPQVLEATAALYNQDLSQLELLLGGLLESHG-DPGPLFSAIVLD 506
drDUOX  422 QRGRDFGLPSYNQIREALSMAPVNSFEDINPKL---KDTKL---ADLYENDISRLELFVGGLLETQE-GPGPVFSTIILD 494
dmDUOX  459 MRGRDNGLPDYNTARESYGLKRHKTWTDINPPLFETQPELLDMLKEAYDNKLDDVDVYVGGMLESYG-QPGEFFTAVIKE 537
ceDUOX  420 MRGRDNGVPPYNELRRTFGLAP-KTWETMNEDFYKKHTAKVEKLKELYGGNILYLDAYVGGMLEGGENGPGELFKEIIKD 498

hDUOX1  504 QFVRLRDGDRYWFENTRNGLFSKKEIEEIRNTTLQDVLVAVINIDPSALQPNVFVWHKGDPCPQPRQLSTEGLPACAPSV 583
hDUOX2  507 QFVRLRDGDRYWFENTRNGLFSKKEIEDIRNTTLRDVLVAVINIDPSALQPNVFVWHKGAPCPQPKQLTTDGLPQCAPLT 586
drDUOX  495 QFERIRNADRFWFENKQNGLFTEEEIKAIRNTTFHDVLLHVTSAEKGDIQRSVFFWKNGDPCPQPQPIRASDLQPCTKAF 574
dmDUOX  538 QFQRLRDADRFWFENERNGIFTPEEIAELRKITLWDIIVNSTDVKEEEIQKDVFMWRTGDPCPQPMQLNATELEPCTYLE 617
ceDUOX  499 QFTRIRDGDRFWFENKLNGLFTDEEVQMIHSITLRDIIKATTDIDETMLQKDVFFFKEGDPCPQPFQVNTTGLEPCVPFM 578

hDUOX1  584 VRDYFEGS 591
hDUOX2  587 VLDFFEGS 594
drDUOX  575 SMSYFDDS 582
dmDUOX  618 GYDYFSGS 625
ceDUOX  579 QSTYWTDN 586
```

Supplementary Fig. 5 | Sequence alignment of the peroxidase homology domain (PHD) of DUOX. The sequences of the *Homo sapiens* DUOX1 (hDUOX1), *Homo sapiens* DUOX2 (hDUOX2), *Danio rerio* DUOX (drDUOX), *Drosophila melanogaster* DUOX (dmDUOX) and *Caenorhabditis elegans* DUOX (ceDUOX) were aligned. Residues following PHD are omitted for clarity. The sequence alignment of Figs. S5-8 are all shown as follows: Conserved residues are highlighted in gray; Secondary structures are indicated as cylinders (α helices), arrows (β sheets) and lines (loops); Unmodeled residues are indicated as dashed line; The color of arrows and cylinders are the same as in Fig. 1**h**. Residues of cation binding site 1 (CBS1) and cation binding site 2 (CBS2) are indicated as black circles and violet circles, respectively. The filled circles and empty circles indicate side chain and main chain interactions.

DUOX M0-TMD

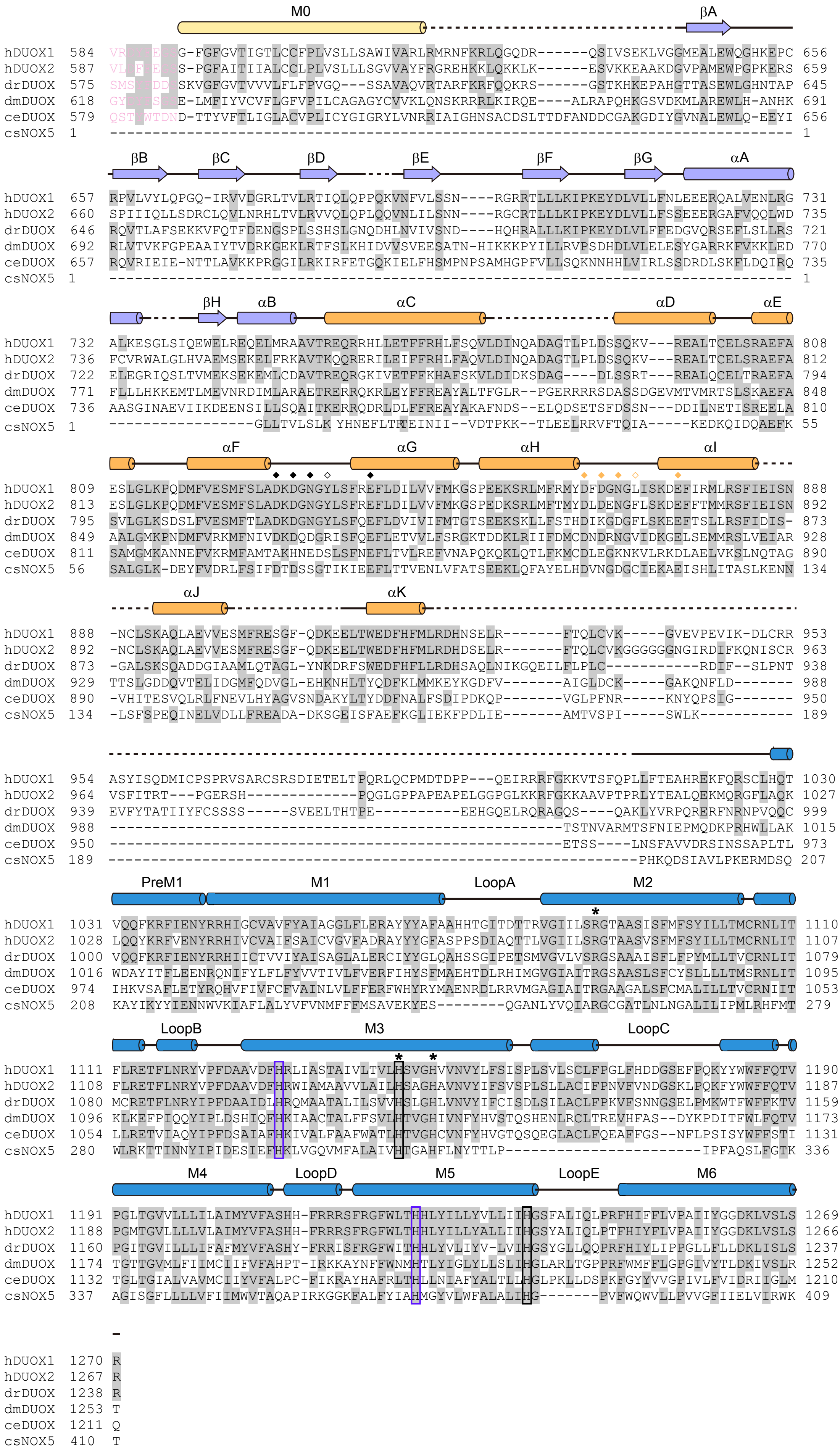

Supplementary Fig. 6 | Sequence alignment from M0 helix to transmembrane domain (TMD) of DUOX. The sequences of the *Homo sapiens* DUOX1 (hDUOX1), *Homo sapiens* DUOX2 (hDUOX2), *Danio rerio* DUOX (drDUOX), *Drosophila melanogaster* DUOX (dmDUOX), *Caenorhabditis elegans* DUOX (ceDUOX) and *Cylindrospermum stagnale* NOX5 (csNOX5) were aligned. Residues belonging to PHD are shown in violet, and residues following TMD are omitted for clarity. Residues predicted to be responsible for calcium binding in EF1 and EF2 are indicated as black diamonds and orange diamonds, respectively. The filled diamonds and empty diamonds indicate side chain and main chain interactions. Histidines coordinating the outer haem and the inner haem are enclosed by black boxes and blue boxes, respectively. Residues of proposed oxygen substrate binding site are indicated as asterisks.

DUOX DH

```
                   β1     β2         β3                β4               β5               β6         α1
hDUOX1  1270  RKKVEISVVKAELLPSGVTHLRFQRPQGFEYKSGQWVRIACLALGT--TEYHPFTLTSAPH-EDTLSLHIRAAGPWTTRL 1346
hDUOX2  1267  RKKVEISVVKAELLPSGVTYLQFQRPQGFEYKSGQWVRIACLALGT--TEYHPFTLTSAPH-EDTLSLHIRAVGPWTTRL 1343
drDUOX  1238  RKKVEIPVLKAELLPSDVTMLEFKRPQGFVYRSGQWVRIACLTLGT--DEYHPFTLTSAPH-EETLSLHIRAAGPWTSKL 1314
dmDUOX  1253  TKYMALDVIDTDLLPSDVIKIKFYRPPNLKYLSGQWVRLSCTAFRP--HEMHSFTLTSAPH-ENFLSCHIKAQGPWTWKL 1329
ceDUOX  1211  QYYKKLEIVNAEILPSDIIYIEYRRPREFKYKSGQWVTVSSPSISCTFNESHAFSIASSPQ-DENMKLYIKAVGPWTWKL 1289
csNOX5  410   TTKEPTFVVNASLLPSKVLGLQVQRPQSFNYQPGDYLFIKCPGISK--FEWHPFTISSAPEMPDVLTLHIRAVGSWTGKL 487

              α1                     β7                  β8         α2
hDUOX1  1347  REIYSAPT-------GDRCARYPKLYLDGPFGEGHQEWHKFEVSVLVGGGIGVTPFASILKDLVFKSSVS--CQVFCKKI 1417
hDUOX2  1344  REIYSSPK-------GNGCAGYPKLYLDGPFGEGHQEWHKFEVSVLVGGGIGVTPFASILKDLVFKSSLG--SQMLCKKI 1414
drDUOX  1315  REAYSPEK-------HQELGGLPKLYLDGPFGEGHQEWTDFEVSVLVGAGIGVTPFASILKDLVFKSSVK--FKFHCKKV 1385
dmDUOX  1330  RNYFDPCN--------YNPEDQPKIRIEGPFGGGNQDWYKFEVAVMVGGGIGVTPYASILNDLVFGTSTNRYSGVACKKV 1401
ceDUOX  1290  RSELIRSL--------NTGSPFPLIHMKGPYGDGNQEWMDYEVAIMVGAGIGVTPYASTLVDLVQRTSSDSFHRVRCRKV 1361
csNOX5  488   YQLIREQREEWIRSGSSQSLPGVPVYIDGPYGTPSTHIFESKYAILICAGIGVTPFASILKSILHRNQQN-PAKMPLKKV 566

              β9          α3              β10            α4                          β11
hDUOX1  1418  YFIWVTRTQRQFEWLADIIREVEENDHQDLVSVHIYITQLAEKFDLRTTMLYICERHFQKVL-NRSLFTGLRSITHFGRP 1496
hDUOX2  1415  YFIWVTRTQRQFEWLADIIQEVEENDHQDLVSVHIYVTQLAEKFDLRTTMLYICERHFQKVL-NRSLFTGLRSITHFGRP 1493
drDUOX  1386  YFLWVTRTQRQFEWLSDIIREVEDMDMQDLVSVHIYITQLPEKFDLRTTMLYVCERHFQKVW-NRSLFTGLRSVTHFGRP 1464
dmDUOX  1402  YFLWICPSHKHFEWFIDVLRDVEKKDVTNVLEIHIFITQFFHKFDLRTTMLYICENHFQRLS-KTSIFTGLKAVNHFGRP 1480
ceDUOX  1362  YFLWVCSTHKNYEWFVDVLKNVEDQARSGILETHIFVTQTFHKFDLRTTMLYICEKHFRATNSGISMFTGLHAKNHFGRP 1441
csNOX5  567   HFYWLNREQKAFEWFVELLSKIEAEDTNNLFDLNLYLTGAQQKSDMKSSTLFVAMDLMHQET-KVDLITGLKSRTKTGRP 645

                α5            β12         α6              β13
hDUOX1  1497  PFEPFFNSLQEVHPQVRKIGVFSCGPPGMTKNVEKACQLINRQ-DRTHFSHHYENF- 1551
hDUOX2  1494  PFEPFFNSLQEVHPQVRKIGVFSCGPPGMTKNVEKACQLVNRQ-DRAHFMHHYENF- 1548
drDUOX  1465  PFLAFLSSLQEVHPEVEKVGVFSCGPPGLTKNVEKACQQMNKR-DQTHFVHHYENF- 1519
dmDUOX  1481  DMSSFLKFVQKKHSYVSKIGVFSCGPRPLTKSVMSACDEVNKTRKLPYFIHHFENFG 1537
ceDUOX  1442  NFKAFFQFIQSEHKEQSKIGVFSCGPVNLNESIAEGCADANRQRDAPSFAHRFETF- 1497
csNOX5  646   DWEEIFKDVAKQHAP-DNVEVFFCGPTGLALQLRHLCTKYG-------FGYRKENF- 693
```

Supplementary Fig. 7 | Sequence alignment of the dehydrogenase domain (DH) of DUOX. The sequences of the *Homo sapiens* DUOX1 (hDUOX1), *Homo sapiens* DUOX2 (hDUOX2), *Danio rerio* DUOX (drDUOX), *Drosophila melanogaster* DUOX (dmDUOX), *Caenorhabditis elegans* DUOX (ceDUOX) and *Cylindrospermum stagnale* NOX5 (csNOX5) were aligned. Residues belonging to TMD are shown in marine. Conserved residues are highlighted in gray. Secondary structures are indicated as cylinders (α helices), arrows (β sheets) and lines (loops). Unmodeled residues are indicated as dashed line. The color of arrows and cylinders are the same as in Fig. 1.

DUOXA

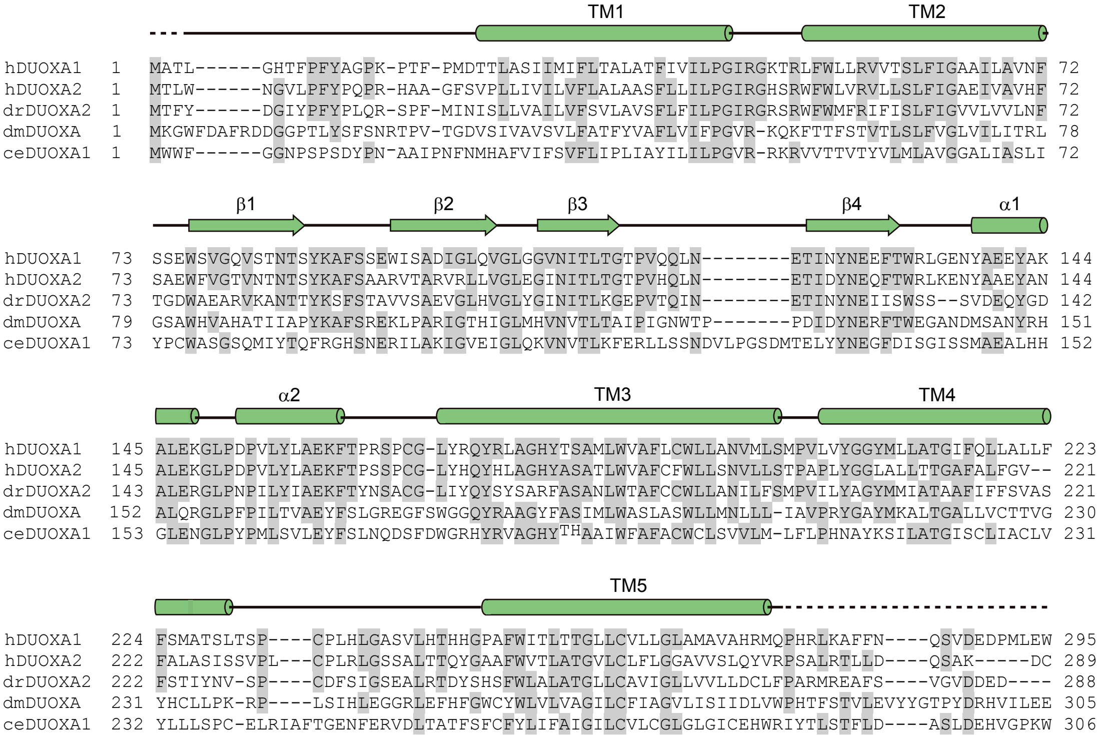

Supplementary Fig. 8 | Sequence alignment of DUOXA. The sequences of the *Homo sapiens* DUOXA1 (hDUOXA1), *Homo sapiens* DUOXA2 (hDUOXA2), *Danio rerio* DUOXA2 (drDUOXA2), *Drosophila melanogaster* DUOXA (dmDUOXA) and *Caenorhabditis elegans* DUOXA1 (ceDUOXA1) were aligned. Conserved residues are highlighted in gray. C-terminal residues are omitted for clarity. Secondary structures are indicated as cylinders (α helices), arrows (β sheets) and lines (loops). Unmodeled residues are indicated as dashed lines. The color of arrows and cylinders are the same as in Fig. 1.

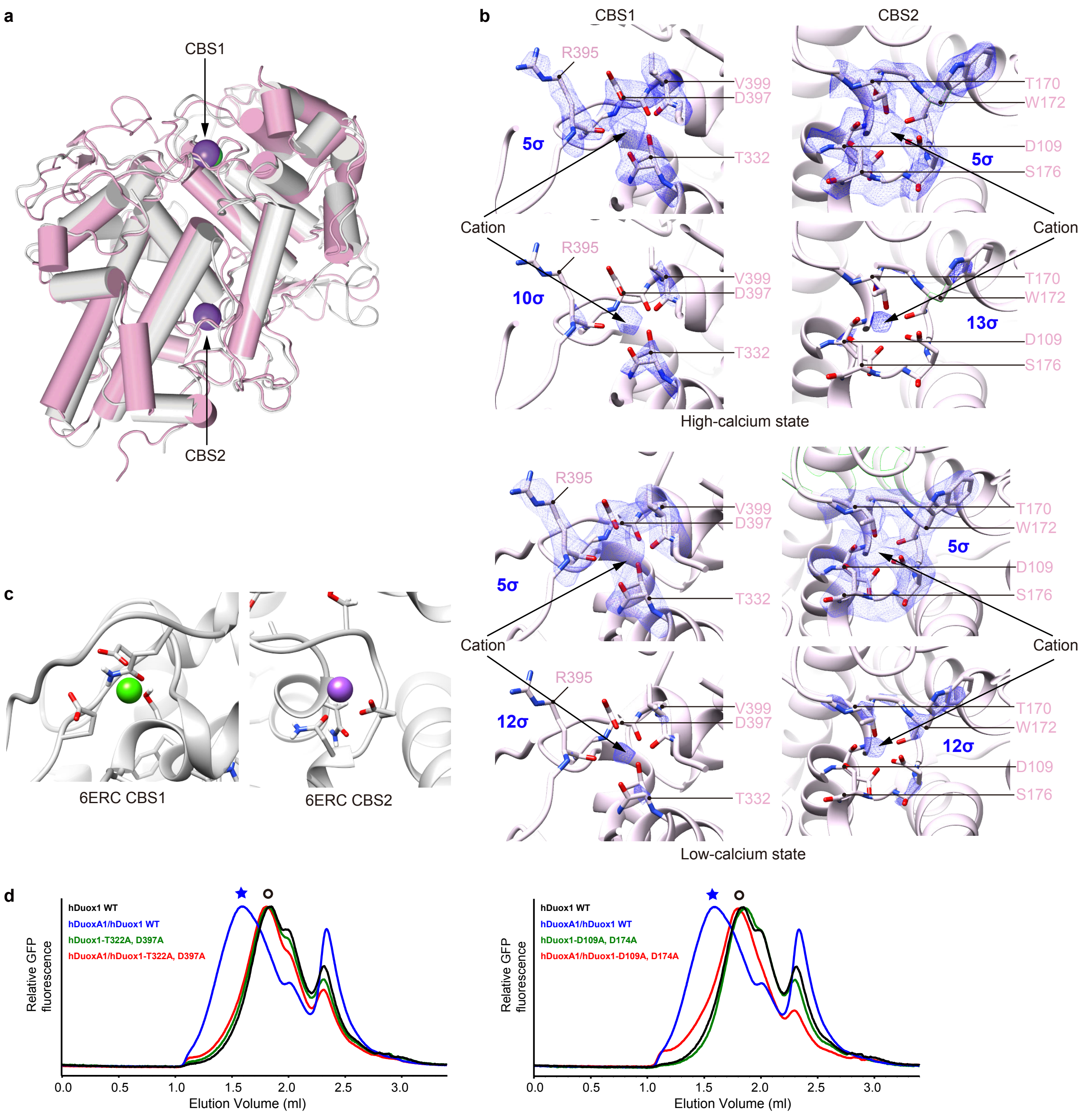

Supplementary Fig. 9 | Structure of hDUOX1 PHD domain. a, Structural comparison between hDUOX1 PHD (pink) and DdPoxA (PDB ID: 6ERC, gray). CBS1 and CBS2 are denoted by arrows. **b**, Electron densities (blue meshes) at the cation bound sites under low (top) and high (bottom) contour level in the high-calcium and low-calcium states. The bound cation densities are indicated by arrows. **c**, CBS1 and CBS2 in DdPoxA (PDB ID: 6ERC). **d**, FSEC profiles showed mutations of hDUOX1 CBS1 (T322A, D379A) or CBS2 (D109A, D174A) affect hDUOX1-hDUOXA1 tetramer assembly. Asterisks denote the peak position of tetrameric complex and circles denote hDUOX1 monomer peak position.

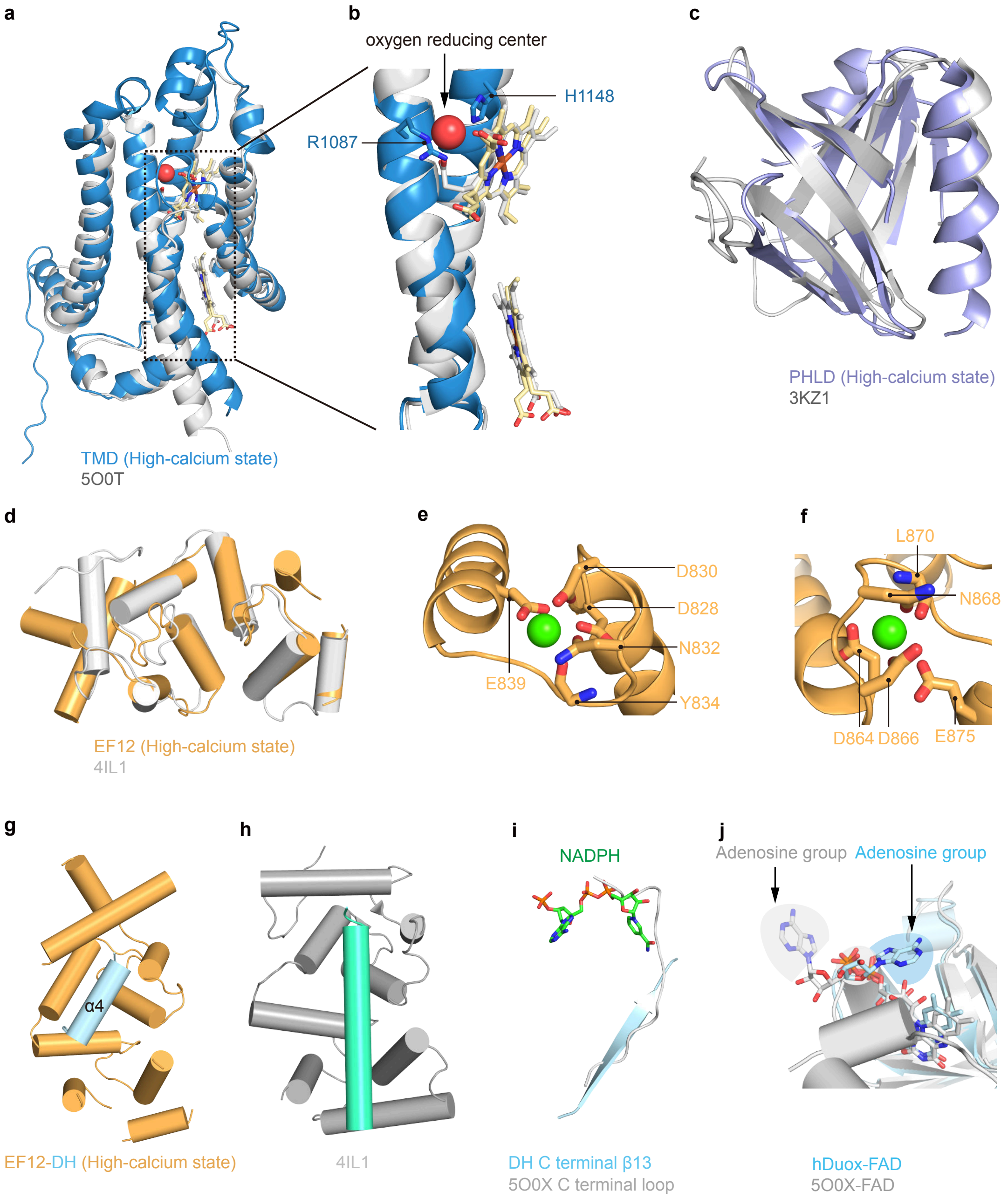

Supplementary Fig. 10 | Structure of hDUOX1 TMD and cytosolic domains. **a**, Structural comparison between hDUOX1 TMD (blue) and csNOX (PDB ID: 5O0T, gray). The putative oxygen-reducing center is shown as red sphere. **b**, The close-up view of the similar architecture of the putative oxygen-reducing center. **c**, Structural comparison between hDUOX1 PHLD (light blue) and PDZ-RhoGEF (PDB ID:3KZ1, gray). **d**, Structural comparison between hDUOX1 EF module (orange) and Calcineurin B subunit (PDB ID: 4IL1, gray). **e**, Calcium binding on EF1 domain. Calcium ion is modeled according to 4IL1 and shown as green sphere. **f**, Calcium binding on EF2 domain. Calcium ion is modeled according to 4IL1 and shown as green sphere. **g**, Structure of hDUOX1 EF module (orange) in complex with α4 helix (light blue) of DH domain. **h**, Structure of calcineurin B subunit in complex with helix of calcineurin A subunit (PDB ID: 4IL1). **i**, Structural comparison of NADPH binding pocket between hDUOX1 (colored) and csNOX (PDB ID: 5O0X, gray). Only β13 and NADPH are shown for clarity. **j**, Structural comparison of FAD binding pocket between hDUOX1 (colored) and csNOX (PDB ID: 5O0X, gray). The large rotations of adenosine group of FAD are denoted by arrows.

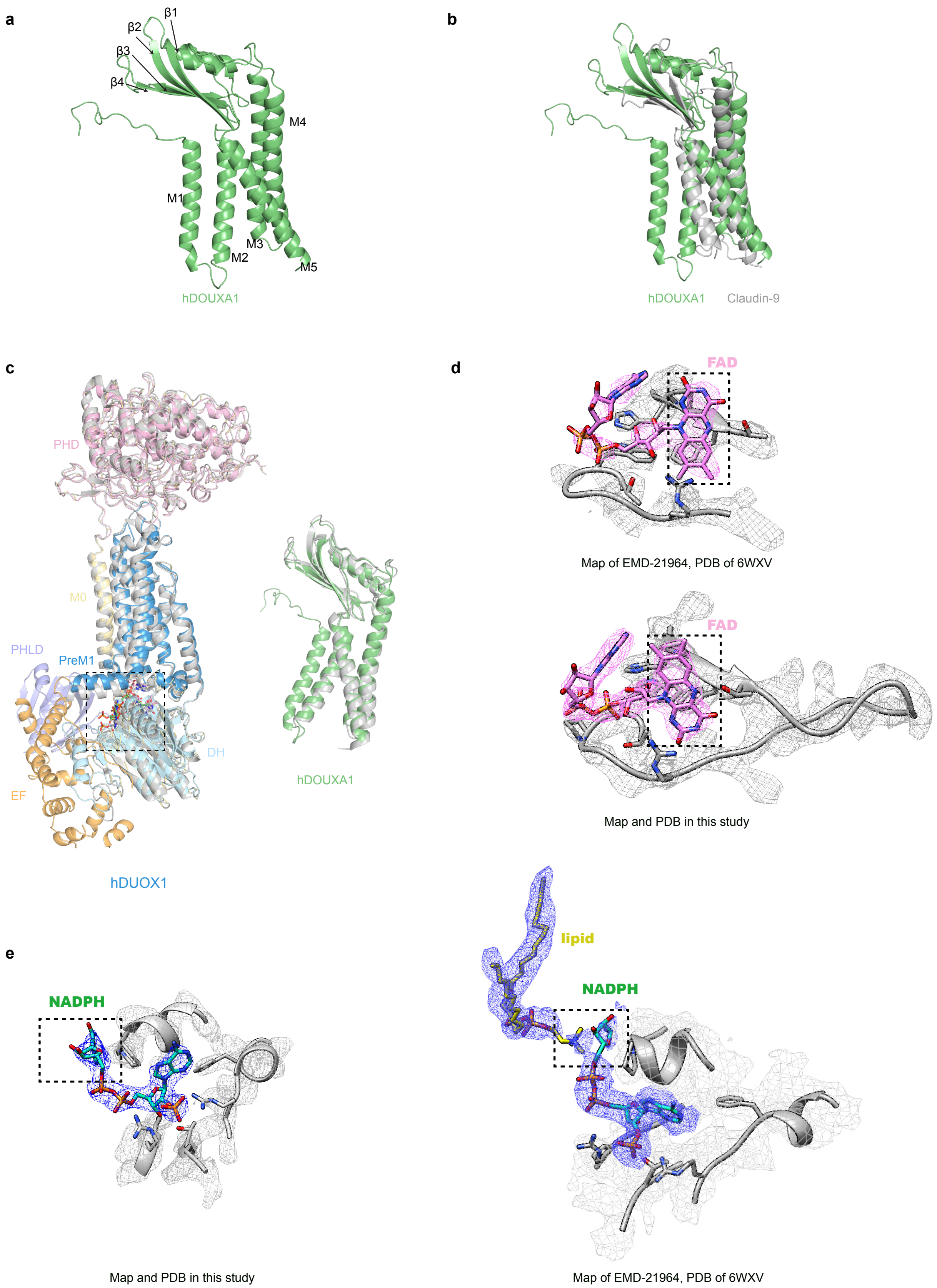

Supplementary Fig. 11 | Structure of hDUOXA1 subunit. a, Structure of hDUOXA1 subunit with secondary structure labeled. **b**, Structural comparison of hDUOXA1 with claudin-9 (PDB ID:6OV2). hDUOXA1 is colored in green and claudin-9 is colored in gray, respectively. **c**, Structural overlay of DUOX1 (left) and DUOXA1 (right) subunits between hDUOX1 in our study (colored) and mDUOX1 published by other group (PDB ID: 6WXV, gray). Dashed lines indicate the substrate binding sites, enlarged in **d-e**. **d-e**, Detailed structural comparison of modeled FAD and NADPH molecules.

Supplementary Table 1 | Cryo-EM data collection, refinement and validation statistics

	High-calcium state	Low-calcium state
PDB ID	7D3F	7D3E
EMDB ID	EMD-30556	EMD-30555
**Data collection and processing**		
Magnification	130,000 ×	130,000 ×
Voltage (kV)	300	300
Electron exposure (e–/Å^2)	50	50
Defocus range (μm)	-1.5 to -2.0	-1.5 to -2.0
Pixel size (Å)	1.045	1.045
Symmetry imposed	*C2*	*C2*
Initial particle images (no.)	1,043,262	783,802
Final particle images (no.)	372,815	125,984
Map resolution (Å)	2.6 (2.5/3.2)*	2.7 (2.8/3.4)*
FSC threshold	0.143	0.143
Map resolution range (Å)	250.0-2.5	250.0-2.8
**Refinement**		
Initial model used (PDB code)	6ERC, 5O0T, 4IL1, 5O0X	
Model resolution (Å)	2.5	2.8
FSC threshold	0.5	0.5
Model resolution range (Å)	250.0-2.5	250.0-2.8
Map sharpening *B* factor (Å^2)	(-98.1/-153.3)*	(-76.0/-122.2)*
Model composition		
Non-hydrogen atoms	26,466	26,012
Protein residues	3,282	3,304
Ligands	46	42
Water	2	2
B factors (Å^2)		
Protein	69.42	113.39
Ligand	61.83	108.21
Water	60.06	109.22
R.m.s. deviations		
Bond lengths (Å)	0.004	0.004
Bond angles (°)	0.927	0.945
Validation		
MolProbity score	1.68	1.45
Clashscore	6.64	6.87
Poor rotamers (%)	2.58	0.44
Ramachandran plot		
Favored (%)	98.24	97.70
Allowed (%)	1.76	2.30
Disallowed (%)	0.00	0.00

* The numbers outside the brackets are from the consensus refinement. Numbers inside brackets are from the multibody refinement (large body/small body).

Supplementary Table 2 | Primers used in this study

Primer Name	Sequene
hDUOX1_21_NGFP_F	CAAGGCCCTGAATTCggagctcagaaccccatt
hDUOX1_FL_C.stop_R	GTGGTGGTGCTCGAGctagaagttctcataatggtg
hDUOXA1_FL_N_F	ATATGAATTCatggctactttggga
hDUOXA1_FL_C.stop_R	ATATCTCGAGttataaagcacaatcaggatctttggggtgtgcctccttacagtatgccttggtggaggaag
hDUOXA1_FL_C_R	CAGGTTTTCCTCGAGtaaagcacaatcagg
hDuox1_R507A_F	cttgaacaatttgtgGCgctacgggatggt
hDuox1_R507A_R	accatcccgtagcGCcacaaattgttcaag
hDuox1_R507E_F	cttgaacaatttgtgGAgctacgggatggt
hDuox1_R507E_R	accatcccgtagcTCcacaaattgttcaag
hDuox1_R50E_F	ggcagcaaaggctccGAgctgcagcgcctg
hDuox1_R50E_R	caggcgctgcagcTCggagcctttgctgcc
hDuox1_T332AD397A_F	gagcagttcctgtccGccatggtgcccccct
hDuox1_T332AD397A_R	aaccaacacatggGcctctcgctctgcgat
hDuox1_D109AD174A_F	tatcacgtgctttcagCcctggtgagcgtg
hDuox1_D109AD174A_R	gatggcgctgccgGccagccagcccgtcac
