# Supplementary material for: Structures of human dual oxidase 1 complex in low-calcium and high-calcium states
Source: Nat Commun. 2021 Jan 8;12:155. doi: 10.1038/s41467-020-20466-9 (PMC7794343; doi:10.1038/s41467-020-20466-9)
Supplement: Supplementary file 2 — Description of Additional Supplementary Files [file 41467_2020_20466_MOESM2_ESM.docx]

Supplementary Movie 1 | Structural changes of the cytosolic domains of hDUOX1-hDUOXA1 complex during calcium activation Description: hDUOUX1-hDUOXA1 complex is shown as cartoon and colored the same as Fig.1h. Calcium ions are shown as green spheres. The movie starts from the whole molecule and then focuses on the cytosolic domains of one protomer. The structural changes are presented as a morph between the high-calcium state and the low-calcium state
